# Cases of Erysipelas

**Published:** 1828-01

**Authors:** 


### ERYSIPELAS.

*Cases of Erysipelas, treated at the* MIDDLESEX HOSPITAL.

WITHIN the last few weeks, a great many cases, both of phlegmonous and idiopathic erysipelas, have been admitted into this hospital; and of these the following have been selected, as exemplifying the variety of treatment which erysipelas requires in some of its different forms.

### CASE I. *Treated with Fomentations.*

Thomas Briant, fifty years of age, a stout athletic porter, was admitted on Friday, the 28th of September, into the hospital, with phlegmonous erysipelas of the hand and forearm. He stated that, five days previously, he received a small punctured wound in the elbow from a spike. This, however, caused him so little uneasiness, that on the next day he went to his usual employment; but, the same afternoon, he felt a good deal of pain in the elbow, which obliged him to leave his work; and in the evening he had a severe rigor. On the Wednesday the arm had become painful, and was slightly swollen; it continued increasing until the Friday, when he came to the hospital.

When admitted, the hand and forearm were considerably swollen, and very tense, yielding to firm pressure, and of a dusky red colour. His pulse was small and exceedingly rapid; his tongue coated with a brown fur; skin hot and dry; and his countenance expressive of great anxiety.

The treatment consisted in warm fomentations of Poppy-heads to the arm, with a purge of Jalap and Calomel.—Hyd. Subm. gr. v.; Ant. Tart. gr. $\frac{1}{4}$; Opii gr. ss. nocte.—Haust. Potassæ supertart. ℥jss.; Vin. Ant. Tart. ʒj. quartis horis.

29th.—His arm is much less tense, and is neither so red nor so painful. He has slept some hours during the night; his bowels are freely open; his tongue is moistening. The erysipelas is extending towards the shoulder. He complains of slight pain in the chest, with some cough.

Continue.

30th.—The arm is much less painful, is not so tense, and has more of its natural colour. The wound of the elbow discharges very freely, and from the appearance of the discharge it is supposed that the wound has penetrated the joint.

Continue.

October 1st.—In every respect much better: the arm is soft, and the erysipelas is disappearing.

Ordered to continue the Salines without the Antimony, and to have five grains of Dover's powder at bedtime.

2d.—He continues improving; and, as all inflammatory symptoms are subdued, he is ordered a more generous diet, with the infusion of Bark, and three drachms of the Liquor Ammoniæ Acetatis three times a day.

From this date he continued daily getting better: the erysipelas left him, the arm recovered its natural size, and the wound ceased discharging; and, on the 9th, he was deemed sufficiently well to leave the hospital, and be made an out-patient.

CASE II. *Treated with Scarifications.*

29th September, 1827.—John Allwright, æt. fifty-three, a poor half-starved labourer, in removing some slates from a barge, about a fortnight previous to his admission, lacerated the little finger of his right hand. He paid little or no attention to it until four or five days back, when the finger became painful, the pain running up to the axilla, and the arm began to swell.

When brought here, he had an ill-conditioned suppurating wound on the little finger; the hand and forearm were prodigiously swollen, of a bluish dusky red, very tense, slightly œdematous in parts, and very painful. There was also a blush of erysipelas extending to the shoulder. His pulse was small, and very rapid (140); his tongue coated with a brown fur; bowels costive; and his countenance flushed, haggard, and very anxious.

Some bands of integuments, which strictured the finger, were divided, and slight scarifications through the skin were made on the hand and arm.—Ordered Poppy fomentations locally.—Hydr. Subm. gr. v.; Ant. Tart. gr. $\frac{1}{4}$; Opii gr. ss. s. et p.—Infus. Sennæ comp. ℥xj.; Magn. Sulph. ʒiij.; Tr. Jalapæ ʒjss. sextis horis donec alvus plene dejic.

30th.—Is somewhat more free from pain, but no decided diminution in the swelling or inflammation.

Liq. An. A. ʒiv.; Magnes. Sulph. ʒijss.; Vin. Ant. Tart. ʒj.; Mist. Camp. ℥viij. sextis horis.

On the 1st of October, he was somewhat better; the arm not so tense nor so painful; bowels freely open; tongue cleaning; pulse softer; and his countenance less anxious.

Ordered to continue the Salines, with Calomel and Dover's powder at bedtime.

From the 1st to the 6th, he continued much in the same condition: the constitutional irritation somewhat abated, and the local disease gradually diminishing.

6th.—He has passed a restless night, and this morning complains of pain in the forearm: it is again much swollen, and the chain of absorbents on the inner side of the arm are greatly inflamed; the hand is swollen and tense; his tongue pasty, and his pulse small, with heat of skin and great irritability.

Scarifications, of an inch in length, were freely made over the hand and arm, and the tension is much relieved.—Ordered to continue the Salines, with three grains of Calomel and five of Dover's powder at bedtime.

8th.—He has passed a restless night, with frequent delirium; his countenance is expressive of great suffering; his pulse very small, and exceedingly frequent; and his tongue coated with a brown fur. Matter had formed at the wrist; and a large and depending opening was made, and about eight ounces of good pus evacuated.

**Ordered nutrients, with the Infusion of Bark and the Liq. An. A.**

From this date to the 31st, he continued improving: his tongue became clean, the bowels were regular, and his pulse quiet and natural, and he was gaining flesh and strength. The discharge from the wrist was good, and less in quantity. Abscesses successively formed in the hand; and these were opened early, and caused little uneasiness.

On the 31st, a patient in the same ward was carried to the theatre to suffer amputation: the sight of his fellow sufferer, brought down pale and blanched, and without his limb, so alarmed his naturally nervous constitution, that erysipelas again attacked the hand and arm. It was ushered in with a rigor, and a dry tongue and rapid pulse. The treatment consisted, as in the former attack, in the exhibition of Calomel and Antimony, with Salines; but his pulse sunk so rapidly under this treatment, that recourse was immediately had to bark and wine. From this attack he slowly recovered, very much emaciated, with the hand considerably swollen, and œdematous; and with a number of small abscesses forming between the fingers.

November 10th.—On this day he complains of his elbow, and matter is forming at the lower part of the joint. An incision was made into it, and about four ounces of matter were discharged. The arm has assumed a dusky brown appearance; the hand continues of the same size, and the thumb has become useless from the destruction of the ligaments of the joint; the matter has found its way under the tendons and theca, and he has lost the power of the muscles. His health is better; he sleeps well; has a clean tongue and natural pulse, and he eats his food with appetite.

**He is taking wine, with two grains of the Sulphate of Quinine in Infus. Gentian comp. three times a day.**

12th.—A consultation has been held this day by the surgeons, and, considering the uselessness of the hand, his thumb being destroyed, the great suffering he must undergo in the attempt to save it, and the improbability of his constitution bearing up against the discharge, amputation has been determined on, and proposed to him.

He has not consented to the operation, consequently he remains much in the same condition; and this day (18th) he has an attack of diarrhœa. The hand and fingers are separately encircled with rollers, and gentle pressure is made on the sinuses. It does not discharge much. The arm has assumed a better colour; and, independently of his diarrhœa, he is much as he was.

15th.—Amputation is again proposed to him, but he dreads the operation, and will not consent to its performance.

25th.—He has been mending, on the whole, since the last report: the hand is somewhat smaller, the abscess at the elbow has healed, and the arm has assumed a more natural hue. He eats and sleeps well, and is altogether much improved.

Amputation was again hinted at, and the uselessness of the limb was pointed out; but the poor fellow was so much alarmed, that he suddenly left the hospital on the 29th of November.

### Case III. *Treated with Leeches and Incisions.*

October 17th, 1827.—Jane Tooler, æt. eighty-one, a robust and healthy old woman, fell, and struck her right elbow against a stone; two days afterwards she applied at this hospital for assistance. She says that the previous evening she had a shivering fit, which was followed by vomiting, and that during the night she was hot and restless, and could not sleep. The hand, forearm, and arm, were greatly swollen, of a dull bluish-red colour, very tense, and slightly œdematous on pressure: she does not, however, complain of pain in it. Her face is flushed and anxious; her skin hot and dry; tongue brown and coated; and pulse very rapid, and exceedingly small. There is a trifling abrasion of the skin at the elbow, covered with a slough.

*Treatment.*—Hirudines xxx. above the inflamed surface.—Fotus Papaveris cum Cataplasm Farinæ vulneri.—Hydrarg. Subm. gr. v.; Antimon. Tart. gr. $\frac{1}{4}$ st.—Ol. Ricini ℥j. post sex horas.—Haust. Potass. Supertart. ℥jss.; Vin. Ant. Tart. ʒss. sextis horis.

In the evening, the leeches had not relieved the tension of the arm, and there was considerable pain. An incision was therefore made, about four inches in length, through the integuments down to the fascia, on the superior and outer surface of the arm, and about eight ounces of blood flowed from the wound.

18th.—She has slept during the night, and expresses herself free from pain; the arm is much less tense. The incision is discharging a sero-purulent matter; the tongue is cleaning; bowels freely open, and pulse softer.

Ordered to continue the Salines, with fomentations to the arm.

She continued daily improving, and on the 22d the erysipelas had entirely vanished, and the wound of the incision was granulating kindly; the arm has regained its natural size, and she expresses herself quite well.

She is ordered Bark and Acid, with meat diet.

The wound filled up with granulations, and on the 7th of November she left the hospital, with the wound healed and the arm quite well.

### Case IV. *Treated first with Antiphlogistics, afterwards with Quina.*

Charles Bennett, fifty-one years of age, was admitted into the Middlesex Hospital with a fatty tumor on the right shoulder, over the deltoid muscle. On the 15th of October, this tumor was removed by Mr. Mayo; and on the 19th it was dressed, and the wound looked well, and was uniting kindly.

22d.—This morning he complains of being very sick, and says that he had a rigor last night, and that he has passed a bad and restless night. His pulse is full and sharp, and his tongue foul. A blush of erysipelas is seen spreading from the wound of the shoulder over the arm and chest.

*Treatment.*—Pulv. Ipecac. ℈j. Ant. Tart. gr. j. statim.—Hydr. Subm. gr. iv.; Ant. Tart. gr. ¼, nocte.—Liq. An. A. ʒij.; V. Antim. Tart. ʒss.; Magn. Sulph. ℥jss.; Mist. Camph. ʒx. sextis horis.

23d.—The erysipelas is spreading over the chest, and has now reached the ribs. He complains of great thirst.

Continue the medicines, with Pulv. Ip. co. gr. v. nocte.

25th.—The erysipelas now extends over the chest, back, and shoulders. He has been taking the Salines and Antimony, with Calomel and Dover's powder at night. His tongue is dry and brown, and his pulse small and rapid.

Continue.

29th.—The erysipelas is ascending the neck, and the whole upper half of the body is fully covered with the inflammation. The tongue continues brown and furred, and the pulse is small and feeble. As it is imagined that antiphlogistic measures have had a fair trial, he is now ordered—

Quininæ Sulph. gr. ij.; Infus. Gent. comp. ℥jss. sextis horis.

November 3d.—The erysipelas has now left the body, and is spreading over the head and face. He continues taking the Quina, with the addition of five grains of the Carbonate of Ammonia three times daily.

7th.—The erysipelas has entirely left the head and face, and he is now only complaining of weakness and debility. The wound has entirely healed, and he is merely kept in the hospital that he may recover his strength prior to his dismissal.

Case V. *Treated on the same principles as the preceding.*

Charlotte Newman, forty-seven years of age, a poor emaciated woman, was admitted on the 2d of October, with an impetiginous eruption on both legs. For this complaint she had the compound Calomel pill at night, and the decoction of Sarsaparilla.

The eruption was disappearing, and the ulcers were fast healing, when she was attacked with a severe rigor on the 25th, followed by sickness of stomach, an inclination to vomit, and general febrile symptoms; a brown and dry tongue, and a small and rapid pulse.

An emetic was given, and in the evening she was much relieved.

On the 26th, the symptoms were somewhat aggravated; and, on looking to the legs, an erysipelatous blush was seen on the right one, commencing at the foot, and ascending upwards.

She was ordered five grains of Calomel, with a quarter of a grain of Antimony, at bedtime; and effervescing aperient Salines, with small doses of Tartarised Antimony, every six hours.—Poppy fomentations to the leg.

On the 29th, the erysipelas had reached the knee of the right leg, and had attacked the left one.

Ordered to continue the Salines, with three grains of Calomel, and ten of Dover's powders, at bedtime.

31st.—The erysipelas had progressively spread up both thighs; the tongue was as dry and as brown as at first; the pulse was feeble, small, and extremely rapid; she was weak and sinking. Antiphlogistic measures, which had been pursued from the first, were now considered not only useless, but prejudicial to the patient; consequently she was ordered six ounces of wine, with beef-tea and other nutrients.

On the 2d of November, she was much improved; the erysipelas was disappearing, the bowels were open, and the pulse became quiet and fuller.

Ordered to continue her wine, with Bark and the Liq. An. A.

5th.—The erysipelas has nearly disappeared. A slough has, however, formed on the outer ankle of the right foot, and the sacrum is becoming tender. The tongue is not so clean, and she has some diarrhœa.

Ordered Dover's powder at night; to omit the Bark; and to take half a drachm of the compound Tincture of Bark in every glass of wine.

10th.—The sloughs are separating; the bowels act regularly; the tongue is again clean, and the pulse natural: she is, however, weak, and much emaciated.

Ordered eight ounces of wine daily; two grains of the Sulphate of Quina made into a pill with some Extract of Conium, every six hours.

18th.—The sloughs have separated from the back and ankle: she is, however, becoming weaker every day.

She has three ounces of brandy, with ten of wine, per diem. Two grains of the Quina, with the Carbonate of Ammonia, three times a day.

This poor woman gradually sunk under the process of sloughing, and died November 23d.

